# Arylsulfatase D is a prognostic biomarker that promotes glioma cells progression through JAK2/STAT3 pathway and M2 macrophage infiltration

**DOI:** 10.3389/fonc.2023.1228426

**Published:** 2023-09-12

**Authors:** Zihan Song, Zijun Zhao, Siyu Zhu, Qianxu Jin, Shiyang Zhang, Zairan Wang, Bowei Shen, Zijian Wang, Zongmao Zhao

**Affiliations:** ^1^ Department of Neurosurgery, The Second Hospital of Hebei Medical University, Shijiazhuang, Hebei, China; ^2^ Spine Center, Sanbo Brain Hospital, Capital Medical University, Beijing, China; ^3^ Department of Neurosurgery, The Fourth Hospital of Hebei Medical University, Shijiazhuang, Hebei, China; ^4^ Department of Neurosurgery, Peking Union Medical College Hospital, Chinese Academy of Medical Sciences & Peking Union Medical College, Beijing, China

**Keywords:** ARSD, glioma, prognosis, M2 macrophages, JAK2/STAT3 pathway

## Abstract

**Background:**

Arylsulfatase D (ARSD) belongs to the sulfatase family and plays a crucial role in maintaining the proper structure of bone and cartilage matrix. Although several researches have revealed the functions of ARSD in tumor progression, the prognostic value of ARSD in glioma and the related mechanisms have not been fully investigated.

**Methods:**

We performed a pan-cancer analysis of ARSD, and investigated the relationship between expression of ARSD and overall survival (OS) in multiple glioma datasets. ROC curves and nomograms were created to investigate the predictive capacity of ARSD. Immune and analysis were conducted to investigate the mechanisms underlying the roles of ARSD in glioma. Glioma tissue samples were collected to verify the expression of ARSD in glioma, while the functions of ARSD were explored using cell experiment. M2 macrophage infiltration assay was used to determine the relation between ARSD and tumor immune microenvironment.

**Results:**

Survival analysis indicated that individuals with high ARSD expression in glioma had a shorter survival time. Cox analysis showed that ARSD had a good ability for predicting prognosis in glioma. Immune analysis suggested that ARSD could regulate immune cell infiltration and affect the Cancer-Immunity Cycle to create an immunosuppressive environment. Combined with cell experiment and bioinformatic analysis, we found that ARSD can promote glioma progression through regulation of JAK2/STAT3 pathway and M2 macrophage infiltration.

**Conclusion:**

Our study found that ARSD can promote glioma development by regulating immune microenvironment and JAK2/STAT3 signaling pathway, which provided a potential therapy target for glioma treatment.

## Introduction

Glioma, as the most prevalent type of malignant brain tumors, poses a significant threat to human health for its high mortality rate (1). It is estimated that the annual incidence of glioma in adults is 6/100,000 (2). Current treatment options for patients with glioma include surgery, chemotherapy and radiation therapy (3). Despite undergoing standard treatment, glioma patients still face challenges in improving their prognosis (4). Most patients with glioma survive less than a year, and only 4% of them live for five years or more (5). The 5th edition of the WHO central nervous system (CNS) Classification tumors brought significant changes to the classification of gliomas, integrating the use of molecular diagnostics into the classification system. According to 2021 WHO classification, adult-type diffuse gliomas are divided into only 3 classifications: Astrocytoma, IDH-mutant (covers from grade 2 to grade 4; includes diffuse astrocytoma, anaplastic astrocytoma, and glioblastoma with IDH mutation in the 2016 classification); Oligodendroglioma, IDH-mutant and 1p/19q-codeleted; and Glioblastoma, IDH-wildtype (6). Moreover, grading no longer entirely depends on tumor histology, since some molecular events, like EGFR gene amplification, TERT promoter mutation, can identify the diagnosis of Glioblastoma without histological evidence (6, 7). Therefore, it is necessary to explore new biomarker for the improvement of glioma diagnosis and treatment.

Sulfatases are a series of proteins which play critical roles in bone and cartilage matrix. Arylsulfatase D (ARSD) belongs to the sulfatase family and is situated on the X chromosome alongside other aromatic sulfate enzymes that share similar characteristics (8). Overexpression of ARSD has been demonstrated to activate the Hippo/YAP pathway, leading to inhibition of TNBC (triple negative breast cancer) cell proliferation and migration. Additionally, ARSD might function as a molecular inhibitor of the ERα signaling pathway by preventing uncontrolled activation of ERα in breast cancer cells (9). Researches indicated that ARSD was abnormally highly expressed in chronic lymphocytic leukemia (CLL) and is a novel prognostic factor for CLL (10). According to Lin’s findings, increased expression of ARSD might contribute to amyloidosis in breast cancer cells, and therefore targeting ARSD could be a potential strategy for treating TNBC or Alzheimer’s disease (AD) (11). However, the roles of ARSD and its molecular mechanism have not been investigated in glioma.

Macrophages are a main component of infiltrated immune cells in glioma tissue, which were related to poor prognosis of glioma. Macrophages can be broadly classified into two subtypes: M1 macrophages and M2 macrophages. There is a general consensus in the scientific community that M1 macrophages have the ability to eliminate tumor cells and defend against pathogen invasion, while M2 macrophages primarily contribute to the promotion of tumor growth, invasion, and metastasis (12). Previous research showed that macrophages were more likely to develop into M2 macrophages for the immunosuppressive microenvironment in glioma (13). Glioma cells can secrete various chemokines to recruit macrophages and regulate the M2 macrophage polarization (14). Hence, therapy against macrophage polarization and recruitment can be a promising treatment for glioma.

conducted a thorough analysis of ARSD using various glioma datasets in this study. Different from previous research, our research investigated the prognostic significance of ARSD in glioma based on the new WHO classification. Further investigation has shown the mechanisms of ARSD for glioma using bioinformatic analysis. *In vitro* experiment showed that ARSD can promote glioma cell proliferation through JAK2/STAT3 pathway and regulate M2 macrophage infiltration.

## Material and methods

### Pan-cancer analysis

We utilized The Human Protein Atlas (https://www.proteinatlas.org) to examine the expression of ARSD in different normal tissues and single cell types. Pan-cancer analysis was carried out using the UCSCXenaShiny (https://hiplot-academic.com) online tool (15). Genetic alteration analysis was conducted using the cBioPortal tool (16).

### Survival analysis

Kaplan-Meier plots were drawn to evaluate the correlation between ARSD expression level and patient prognosis. The Survminer package was used for Kaplan-Meier analysis in R. Univariate and multivariate Cox analysis were developed to estimate whether it was an independent risk factor for glioma patients. The data of nine glioma cohorts (TCGA, CGGA, Gravendeel, Rembrandt, Kamoun, Murat, LeeY, Phillips, Freije) was obtained from the Gliovis platform (17), including mRNA expression and clinical information. The sample size of the nine glioma was listed in **Table 1**.

**Table 1:** The sample size of nine glioma cohorts.

Dataset	Sample size
CGGA	1013 samples
TCGA	667 samples
Rembrandt	444 samples
Gravendeel	276 samples
Kamoun	180 samples
LeeY	191 samples
Phillips	100 samples
Freije	85 samples
Murat	80 samples

### Establishment of prognostic model

The prognostic significance of ARSD in glioma was analyzed using Receiver Operating Characteristic (ROC) curves through SurvivorROC package (18). In order to predict the OS in gliomas, nomograms were established using various prognostic factors including clinical features and ARSD expression in TCGA, CGGA and Gravendeel. We then confirmed whether the actual and predicted OS were consistent over 1, 3 and 5 years using calibration curves.

### Immune infiltration analysis

We used the R package “GSVA” to produce enrichment scores for 24 immune cell types (19). The gene sets of the immune cells were downloaded from a previous study (20). The enrichment scores reflected the relative levels of each immune cell type. In 2013, Chen and Mellman introduced the Cancer-Immunity Cycle (21), which clarified the process of killing tumor cells in immune system, including release of tumor cell antigen, presentation of tumor antigen, priming and activation, recruitment of T cells into tumor, infiltration of T cells into tumor, recognition of tumor cells by T cells, and killing of tumor cells (22). Thus, the body can effectively kill tumor cells through the Cancer-Immunity Cycle. Immunotherapy can enhance the functions of immune system for killing tumor cells by amplifying or activating the Cancer-Immunity Cycle. Based on this point, we estimated the associations between ARSD and Cancer-Immunity Cycle in TCGA.

### Enrichment analysis

The TCGA glioma patients were separated into two cohorts (high-ARSD and low-ARSD) using the median ARSD expression as a cutoff. The “limma” R package was utilized to acquire the differentially expressed genes (DEGs). The screening criteria are *p*-adj < 0.05 and |logFC|>1.5. To investigate the mechanism of ARSD in glioma, the “ClusterProfiler” was utilized to conduct gene set enrichment analysis (GSEA) (23).

### Cell culture and transfection

The glioma cell lines (U251, U87, A172, and LN229) and the HA (Human astrocyte) cell line were supplied from the Chinese Academy of Sciences’ Cell Bank. The glioma cells were cultured in DMEM (Biological, Salem, US) with 10% Gibco FBS (GIBCO, US) and maintained in a 37 °C, 5% CO_2_ environment. The siRNA of ARSD was obtained from Han Biotechnology (Shanghai, China) and was transfected into cells using Lipofectamine 3000 (L3000015, Invitrogen). The ARSD siRNA sequences were: 5′- GGUUGCUACGGGAACAAUATT− 3′ and 5′- UAUUGUUCCCGUAGCAACCTT − 3′. The ARSD overexpression plasmid (pcARSD) was constructed from Han Biotechnology (Shanghai, China). After 48h-72h, the transfected cells were harvested for further analysis.

### RT-qPCR

RNA was extracted from HA and U251, U87, A172, LN229 cell lines, using Superbrilliant test (Zhongshi, Tianjin, China). Reverse transcription was performed using the Takara kit (Bori Medical, Beijing, China), while PCR analysis was carried out using SYBR qPCR Mix kit (Vazyme, Nanjing, China). The primer sequences: ARSD:5′-CTACTCTTCACTGTGCAGTCTC-3′, 5′-GAGATGACATTGAAGGCCTTGA-3′. GAPDH: 5′-GGAGCGAGATCCCTCCAAAAT-3′, 5′-GGCTGTTGTCATACTTCT CATGG-3′.

### Immunohistochemical analysis

Paraffin sections from 12 glioma patients were obtained from surgically resected gliomas and classified by senior physicians in the department of pathology in our hospital according to WHO classification (WHO I: 3 samples, WHO II: 3 samples, WHO III: 3 samples, WHO IV: 3 samples). Paraffin sections were antigen-repaired using 1% sodium citrate (Bioss, Beijing, China), and incubated overnight with 0.6% ARSD antibody (SAB, China) at 4°C. The sections were incubated with Antirabbit IgG (Bioss, Beijing, China), and dripping with horseradish labeled chain enzyme ovalbumin working solution.

### Western blot

Protein extraction from cells was performed using protein extraction reagent (K1015, APExBIO), and the concentration was measured using the BCA (bicinchoninic acid). The samples were segregated using denaturing polyacrylamide gel electrophoresis, and the protein bands were subsequently transferred onto polyvinylidene fluoride membranes. The polyvinylidene fluoride membranes were blocked with blocking solution for 15 minutes, after discarding the blocking solution, the membranes were incubated with primary antibody overnight at 4°C. The primary antibodies were diluted in the following proportions: anti-ARSD (1: 1,200), anti-CD68 (1:1,000), anti-CD163 (1:1,200), anti-CD206 (1:1,200), anti-CD115 (1:1,000), anti-PPARG (1:1,000), anti-JAK2 (1:1,500), anti-P-JAK2 (1:1,500), anti-STAT3 (1:1,200), anti-P-STAT3 (1:1,200), and anti-GAPDH (1:10,000). Secondary Antibody (1: 10,000) was incubated for 2 hours away from the light. Infrared imaging scanning instruments were used to detect membranes (Odyssey LI-COR, USA).

### CCK8 assay

Logarithmic growth stage U251 and U87 cells were planted into a 96-well plate (100μl 5×10^3^/well). The cells were transfected and then cell viability determined using CCK8 kit at 0d, 1d, 2d, 3d and 4d respectively after transfection. Finally, we added CCK8 (10μl) (Dojindo, Shanghai, China) to each well and measured absorbance at 450 nm measured after 2 hours. (SpectraMax Plus 384).

### Colony assay

The glioma cells were planted into the six-well plate at 1000 cells/well incubated for two weeks. The cells were treated with 4% paraformaldehyde, followed by staining with 0.1% crystal violet. The number of colonies was then quantified by analyzing the images using Image J software (version 1.52 2p).

### Transwell experiment

The experiment was divided into two groups: 1. coated matrix gel group to detect cell invasion ability. 2. uncoated matrix gel to detect cell migration ability. Adding 1×10^5^ U251 or U87 cells and 300μl of serum-free medium to the upper chamber. The lower chamber received 700 µl of medium containing serum. Fixation and staining were performed at 24 hours, in the same way as above.

### Wound healing experiment

The U251 and U87 cells were planted into a 6-well plate for 24h. A pipette tip was employed to create a scratch on the cells, forming a slit. Images of the scratches were taken at the same position at 0, 24, and 48 hours using a camera fitted microscope (Olympus, Japan). To assess the cells’ wound healing capability, the width of the scratch was measured at various time points using Image J software.

### M2 macrophage infiltration assay

Preparation for polarization of M2 macrophages: THP-1 cells were induced to differentiate into macrophages by 100ng/ml PMA (Beyotime; shanghai, China) for 24 h. RT-qPCR and Western blot was used to verify M0 markers CD68. Macrophages were polarized with 20ng/ml IL-4(Nearshore proteins; Suzhou; China) and 20ng/ml IL-13(Nearshore proteins; Suzhou; China) for 48h in the presence of PMA. Then RT-qPCR and Western blot was used to identify the M2 macrophage markers CD163, CD206, CD115 and PPARG.

For M2 macrophage infiltration test, in the upper chamber of Transwell plate, 5.0×10^4^ M2 macrophages were inoculated and 300μl of serum-free 1640 medium was added. A total of 5.0×10^4^ U251 and U87 cells were seeded in the lower chamber, and 700μl of serum-containing DMEM medium was added. Fixation and staining were performed at 24 hours, in the same way as above.

## Results

### Analysis of sulfatase family in TCGA glioma cohort

We explored the expression level of sulfatase family in TCGA glioma cohort and found that most of sulfatase family genes were differently expressed between glioma and normal tissues (**Supplemental Figure 1A**). Univariate and multivariate Cox analysis were performed based on the expression of sulfatase family, and the result showed that ARSD was significantly related to the prognosis of glioma (**Supplemental Figure 1B**). Survival analysis in TCGA LGG and TCGA GBM indicated that high expression of ARSD tended to have a poor prognosis (**Supplemental Figure 1C**).

### Pan-cancer analysis

**Figure 1:**
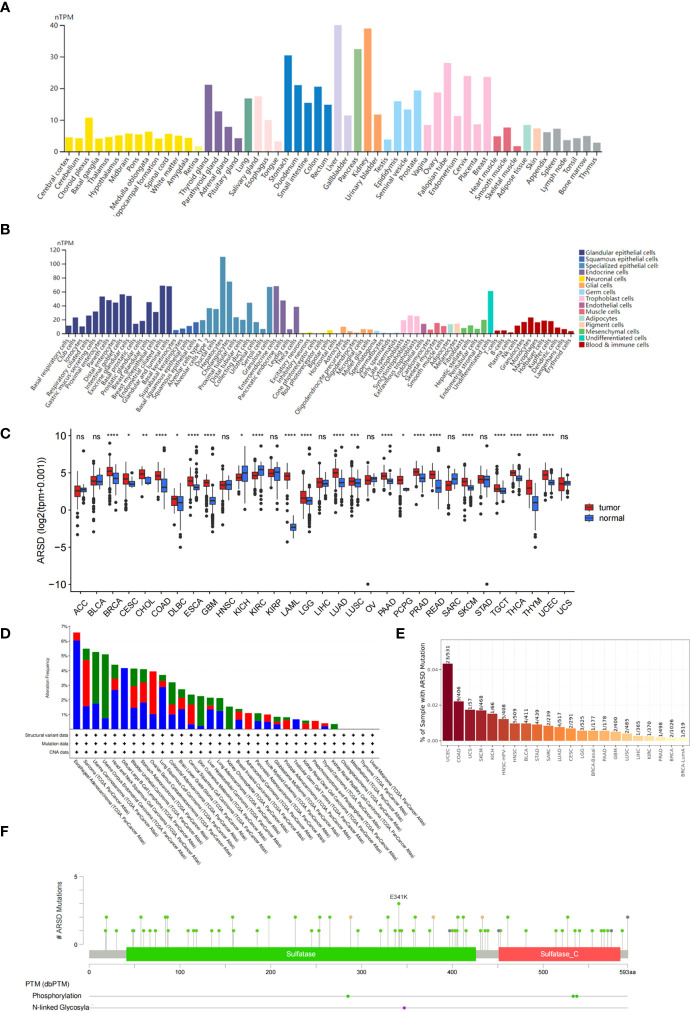
Expression level and mutation analysis of ARSD. **(A)** The expression level of ARSD in normal tissues. **(B)** Expression of ARSD in different single cell types. **(C)** The expression of ARSD in TCGA pan-cancer. **(D)** ARSD alterations occur in different tumor types. **(E)** TIMER analysis of ARSD mutation rates. **(F)** Analysis of ARSD mutations in cBioPortal. * *p* < 0.05 , ** *p* < 0.01, *** *p* < 0.001, ns, no significance.

According to the GTEx database, ARSD is expressed in multiple normal tissues, including the brain, stomach, and liver (**Figure 1A**). In addition, ARSD is also expressed in many single cell types, such as epithelial cells and neuronal cells (**Figure 1B**). Pan-cancer analysis demonstrated that ARSD was aberrantly expressed in most cancer types of TCGA (**Figure 1C**). ARSD alterations occur in a variety of tumors, such as deep in esophageal adenocarcinoma deletion, mutation in UCEC and GBM (**Figure 1D**). TIMER analysis revealed that the top 3 tumors with ARSD mutation rates were UCEC (23/531), COAD (9/406), and UCS (1/57) (**Figure 1E**). Analysis in cBioPortal found that ARSD mutations were mainly concentrated on E341K (**Figure 1F**).

### Survival and expression analysis

**Figure 2:**
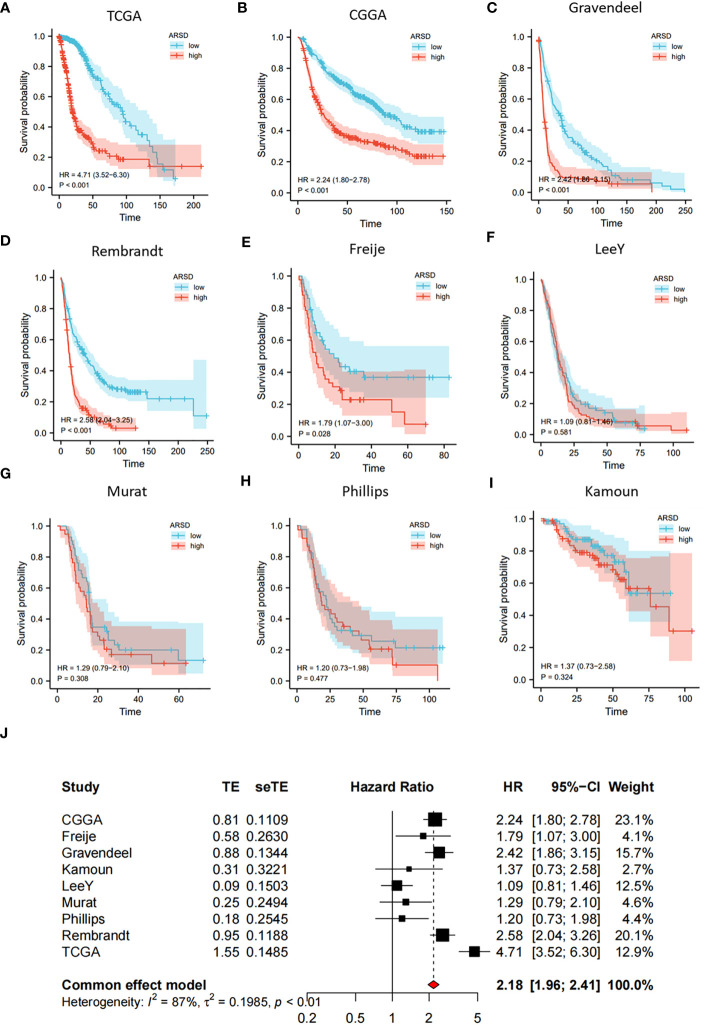
Increased expression of ARSD predicted poor OS for glioma. **(A-I)** Survival analysis in nine glioma populations. **(J)** The total HR value of ARSD was gathered using meta-analysis.

We performed survival analysis in nine glioma cohorts: CGGA (HR:2.24, *p*<0.001), TCGA (HR:4.71, *p*<0.001), Rembrandt (HR:2.58, *p*<0.001), Gravendeel (HR:2.42, *p*<0.001), Kamoun (HR:1.37, *p*=0.324), Freije (HR:1.79, *p*=0.028), LeeY (HR:1.09, *p*=0.581), Murat (HR:1.29, *p*=0.308) and Phillips (HR:1.20, *p*=0.477) (**Figures 2A-I**). Although the results of LeeY, Murat, Phillips, and Kamoun showed no significant difference in p-values, we thought the reason might be the small sample in the four cohorts. To figure out the prognostic value of ARSD in glioma, we performed a meta-analysis based on HRs of the nine glioma cohorts (**Figure 2J**).

The 2021 WHO CNS tumors Classification enhanced our understanding of glioma. We performed survival analysis in different glioma subtypes based on the new WHO Classification. The results showed that ARSD had a good prognostic value in astrocytoma (IDH-mutant, covers from grade 2 to grade 4; includes astrocytoma, anaplastic astrocytoma, and glioblastoma in the 2016 WHO classification), glioblastoma (IDH-wildtype); and oligodendroglioma (IDH-mutant and 1p/19q-codeleted) (**Supplemental Figures 2A-I**). Although the p-value in oligodendroglioma showed no significant difference, we thought the reason might be the small sample size and the long survival time of oligodendroglioma.

**Figure 3:**
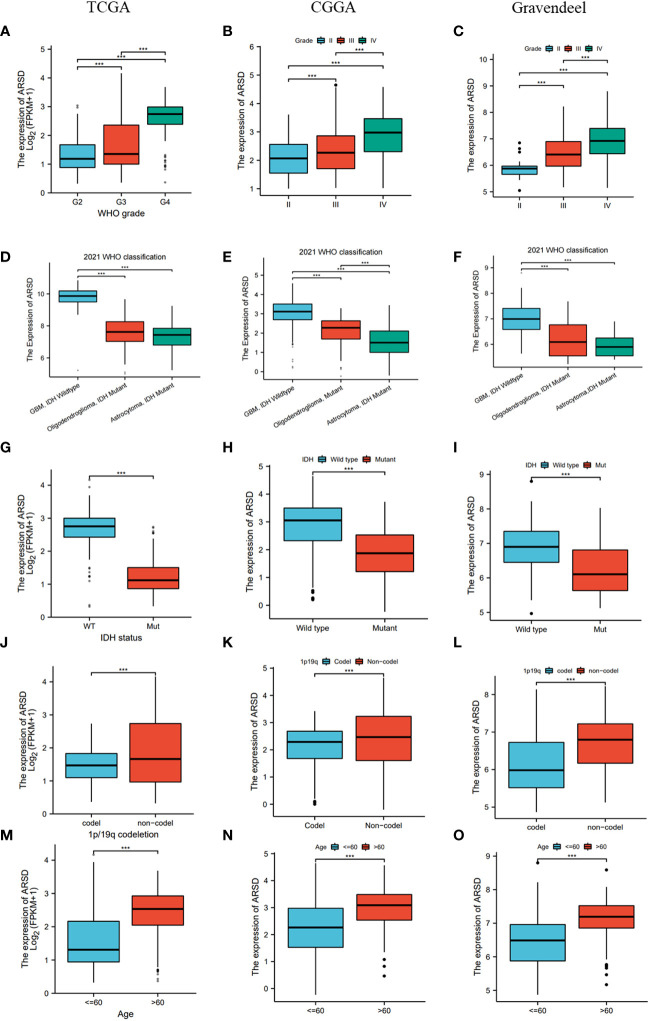
Correlation analysis between ARSD and clinical features in TCGA, CGGA and Gravendeel cohort. **(A-C)** WHO grade; **(D-F)** 2021 WHO classification; **(G-I)** IDH status; **(J-L)** 1p19q status; **(M-O)** Age. *** *p* < 0.001.

Then TCGA, CGGA, and Gravendeel databases was utilized to explore the relation between ARSD and clinical features. We observed that ARSD expressed differently in groups with different WHO grades, IDH status, 1p19q status and age (**Figures 3A-O**).

### Cox analysis and nomogram development

**Figure 4:**
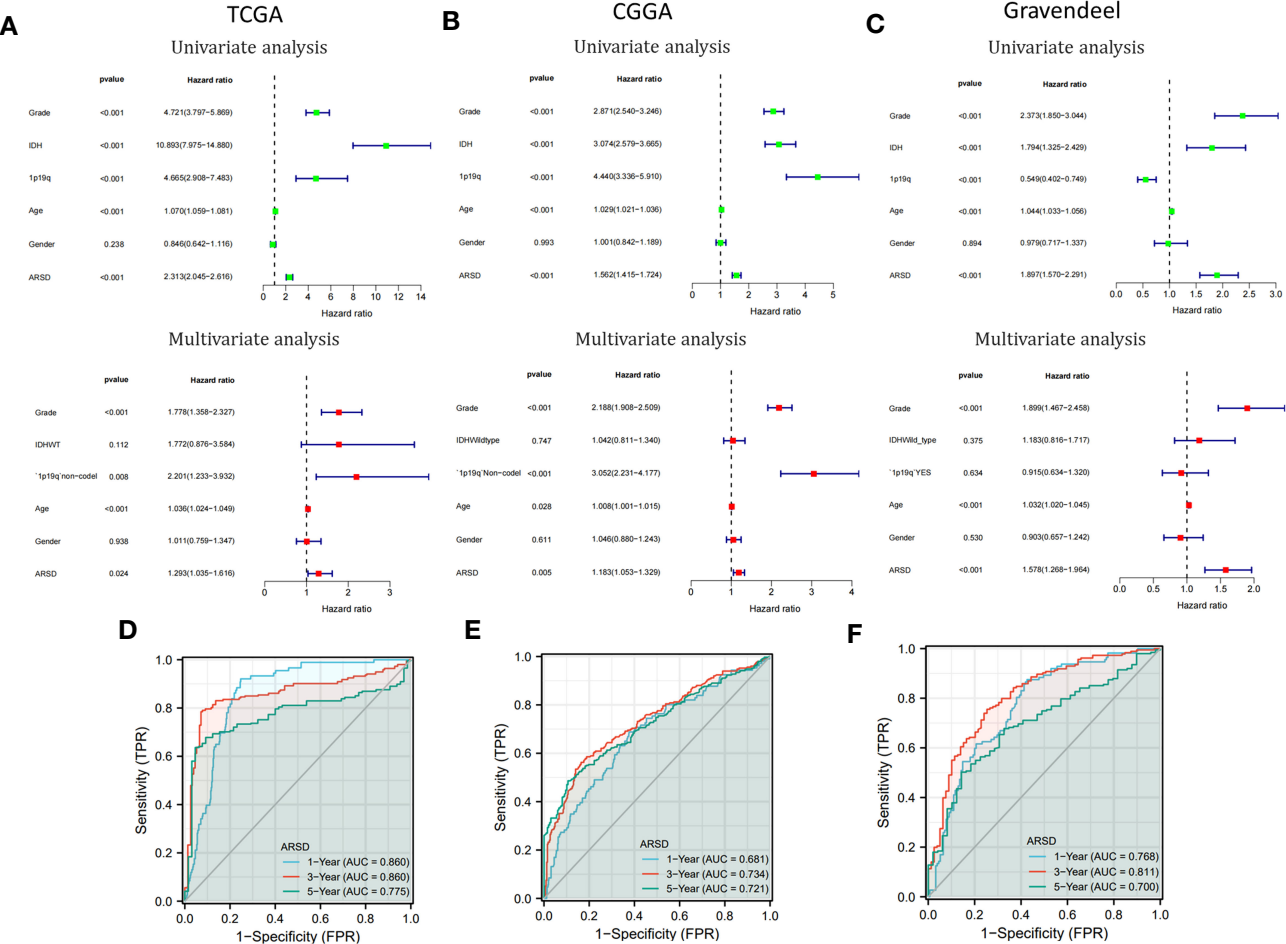
Cox analysis and ROC curves. **(A-C)** Univariate and multivariate analyses of ARSD were conducted using data from TCGA, CGGA, and Gravendeel datasets. **(D-F)** ROC curves of ARSD for predicting 1, 3 and 5year survival in TCGA, CGGA and Gravendeel.

Cox analysis demonstrated that ARSD may serve as a glioma independent prognostic factor (**Figures 4A-C**). ROC curves demonstrated a good ability of ARSD in predicting glioma prognosis at 1, 3 and 5 years (**Figures 4D-F**).

To provide a clinically relevant quantitative method for assessing OS in glioma, we developed a personalized nomogram that combined ARSD with other clinical indicators (TCGA: **Supplemental Figure 3A**; CGGA: **Supplemental Figure 3D**; Gravendeel: **Supplemental Figure 3G**). Decision curves were generated to evaluate the clinical utility of the two nomograms (TCGA: **Supplemental Figure 3B**; CGGA: **Supplemental Figure 3E**; Gravendeel: **Supplemental Figure 3H**). Then we found the calibration curves of the nomogram was basically consistent with the standard curves in one, three, and five-years OS (TCGA: **Supplemental Figure 3C**; CGGA: **Supplemental Figure 3F**; Gravendeel: **Supplemental Figure 3I**).

### The expression of ARSD leads to an immunosuppressive microenvironment

**Figure 5:**
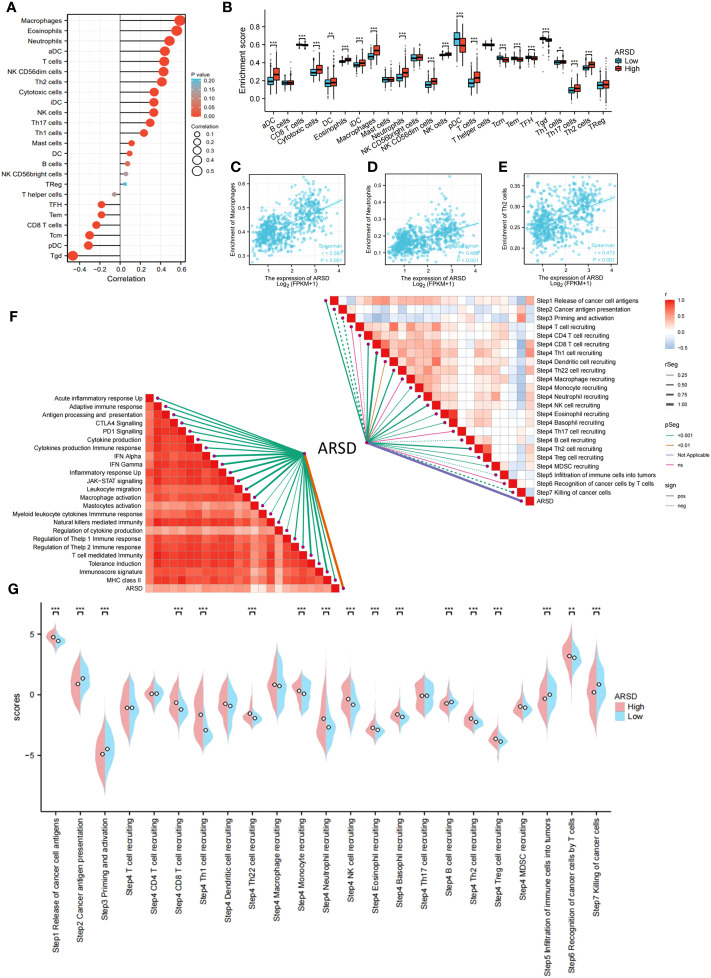
Relation between ARSD and glioma immune microenvironment. **(A)** The levels of 24 immune cell types in high- and low-ARSD groups. **(B)** ARSD expression is correlated with various immune cell types. **(C-E)** The correlation between ARSD expression and infiltration of macrophages **(C)**, neutrophils **(D)** and Th2 cells **(E)**. **(F)** Association between ARSD and innate immunity, adaptive immunity and Cancer-Immunity Cycle. **(G)** A barplot was utilized to display the enrichment scores of the Cancer-Immunity Cycle in the high-ARSD and low-ARSD groups. * *p* < 0.05 , ** *p* < 0.01, *** *p* < 0.001.

The abnormality in tumor immune microenvironment is the main reason for the proliferation, metastasis, and immune evasion of tumor cells. Our findings indicated that ARSD expression was positively correlated with immunosuppressive cells, including M2 macrophages, neutrophils, and Th2 cells (**Figures 5A, B**). Correlation between ARSD and M2 macrophage, Neutrophil, and Th2 cells was analyzed (**Figures 5C-E**). Anti-tumor immunity was conceptualized and proposed as a series of steps called Cancer-Immunity Cycle. Our work revealed the roles of ARSD in anti-tumor immunity of glioma, and found that ARSD expression exhibited a positive correlation with the scores of Step4 and Step1 in the Cancer-Immunity Cycle, while displaying a negative correlation with the scores of Step7, Step5, Step3, and Step2 (**Figures 5F, G**). An analysis of ARSD and 23 types of immune response was then performed to determine whether gliomas exhibit hot immunophenotypes. The related gene signatures were from earlier research (24). We found as the expression of ARSD enhanced, the immunophenotypes trended toward “hot” (**Figure 5F**). These findings suggested that ARSD might regulating Cancer-Immunity Cycle and immune infiltration, thus led to an immunosuppressive microenvironment.

### Enrichment analysis

**Figure 6:**
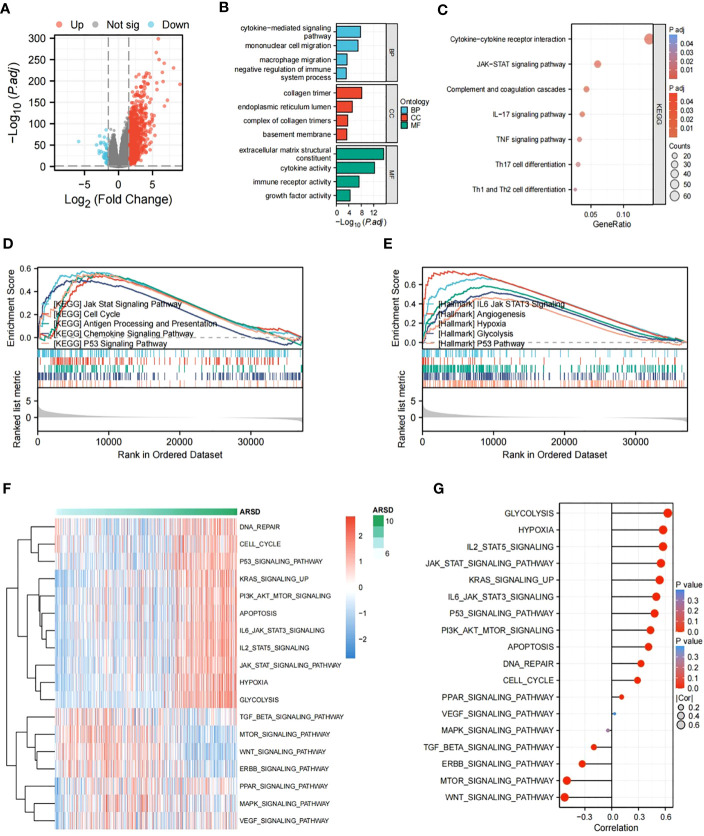
Identification of DEGs and subsequent enrichment analysis were performed. **(A)** The DEGs between high-ARSD and low-ARSD groups were analyzed. **(B)** GO enrichment analysis of DEGs. **(C)** KEGG enrichment analysis of EDGs. **(D, E)** GSEA analysis between high-ARSD and low-ARSD group. **(F, G)** The relation between ARSD and 18-cancer related pathways.

To figure out potential mechanisms of ARSD in glioma, DEGs were explored between low-ARSD and high-ARSD patients in TCGA, where 1,135 DEGs were obtained, containing 46 downregulated genes and 1,089 upregulated genes **(** **Figure 6A**). GO and KEGG analysis revealed that the DEGs were enriched in macrophage migration, negative regulation of immune system processes, JAK-STAT signaling pathway, TNF signaling pathway, and Th1/Th2 cell differentiation (**Figures 6B, C**). The GSEA analysis demonstrated that the JAK-STAT signaling pathway, angiogenesis, glycolysis, and hypoxia were upregulated in patients with high ARSD (**Figures 6D, E**). The GSVA analysis was utilized to explore the relationship between ARSD and 18 tumor-related pathways. The results showed that ARSD was closely related to multiple cancer-related pathways (**Figures 6F, G**).

### ARSD promotes glioma cell proliferation, migration and invasion *in vitro*

**Figure 7:**
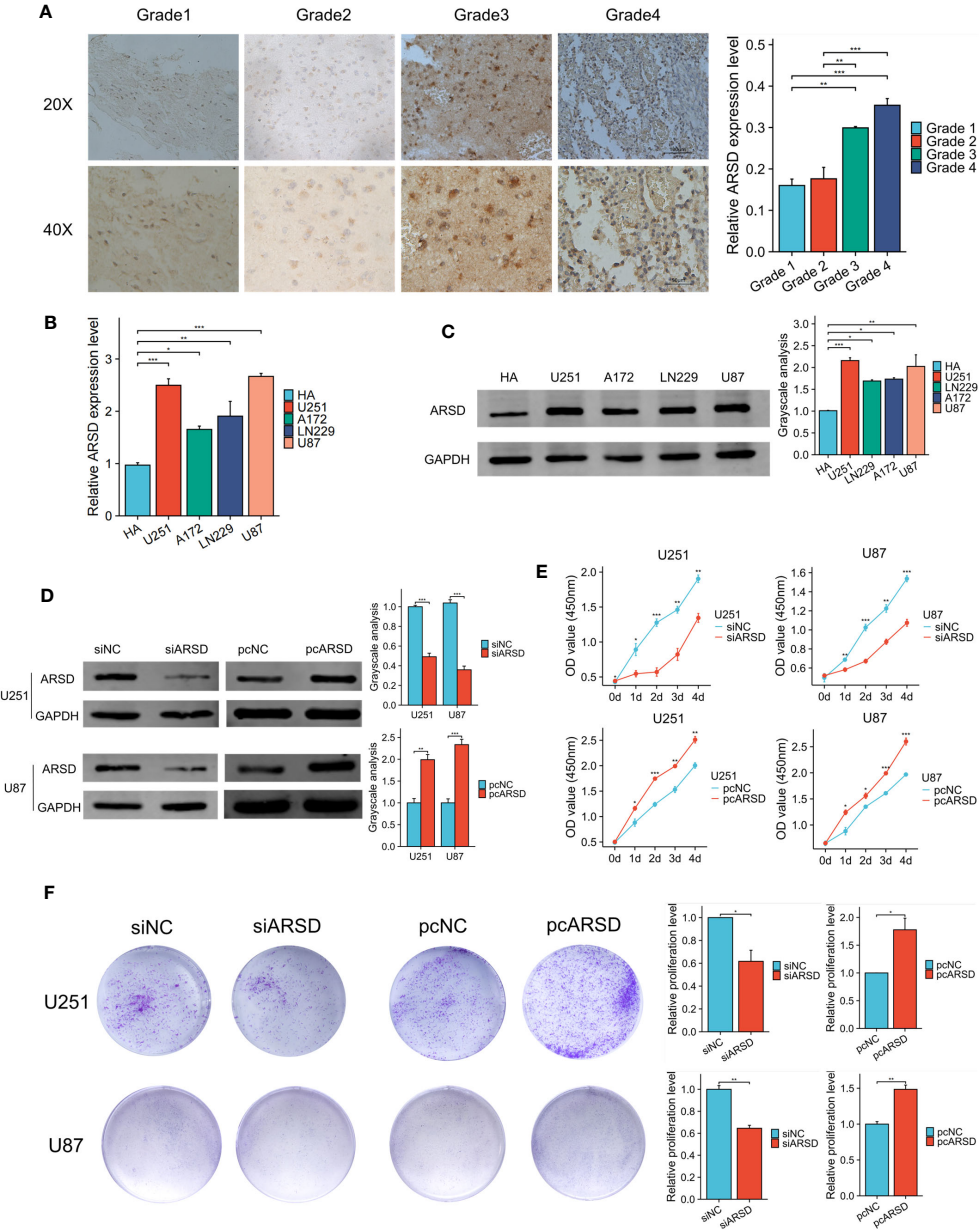
ARSD promotes the proliferation of glioma cells. **(A)** Immunohistochemical images showed the expression of ARSD in different grades of glioma tissue. **(B, C)** PCR and western blot showed the levels of ARSD in HA cells and 4 glioma cell lines. **(D)** The expression level of ARSD was knockdown and overexpressed by using siARSD and pcARSD in glioma cells. **(E, F)** CCK-8 and colony formation assays demonstrated that the glioma cells proliferation was regulated by ARSD. * *p* < 0.05 , ** *p* < 0.01, *** *p* < 0.001.

The immunohistochemical analysis revealed the increase of ARSD expression with higher tumor grades (**Figure 7A**). It is indicated that the level of ARSD expression was higher in U251, U87, A172 and LN229 cell lines compared to HA (**Figures 7B, C**).

**Figure 8:**
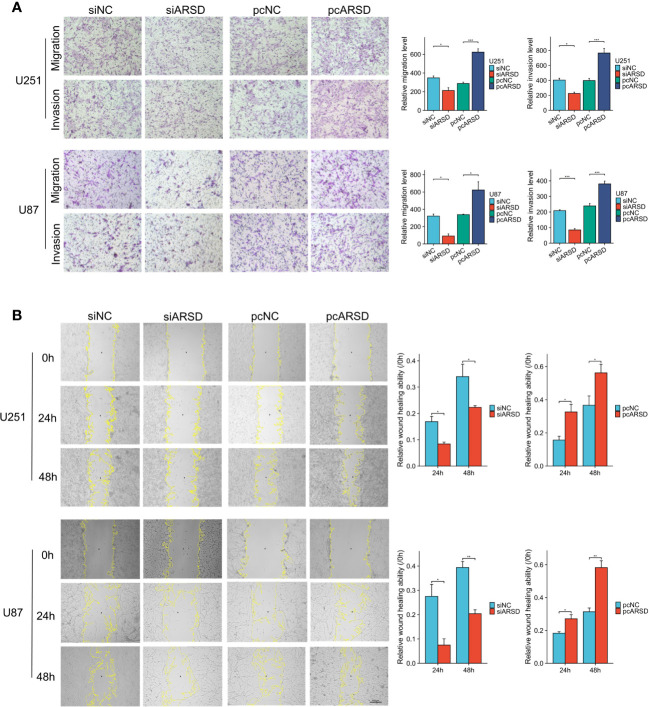
ARSD promotes the migration and invasion of glioma cell lines. **(A)** The upregulation of ARSD expression was found to promote the migration and invasion of glioma cells, as demonstrated by Transwell assay. **(B)** Wound healing assay illustrated that the relative wound healing ability was reduced or increased after knockdown or overexpression of ARSD. * *p* < 0.05 , ** *p* < 0.01, *** *p* < 0.001.

We then performed cell experiment to investigate the roles of ARSD in glioma. Western blotting demonstrated that the expression level of ARSD was lower in U251 and U87 cells transfected with siRNA and higher with plasmids (**Figure 7D**). Both the CCK-8 and colony formation assays demonstrated that knockdown of ARSD significantly suppressed cell proliferation in U251 and U87 cells. Conversely, overexpression of ARSD significantly enhanced cell proliferation (**Figures 7E, F**). The knockdown of ARSD resulted in decreased invasion and migration abilities of U251 and U87 cells, whereas overexpression of ARSD enhances their invasion and migration abilities (**Figures 8A, B**).

### ARSD promotes the infiltration of M2 macrophages regulated by glioma cells

**Figure 9:**
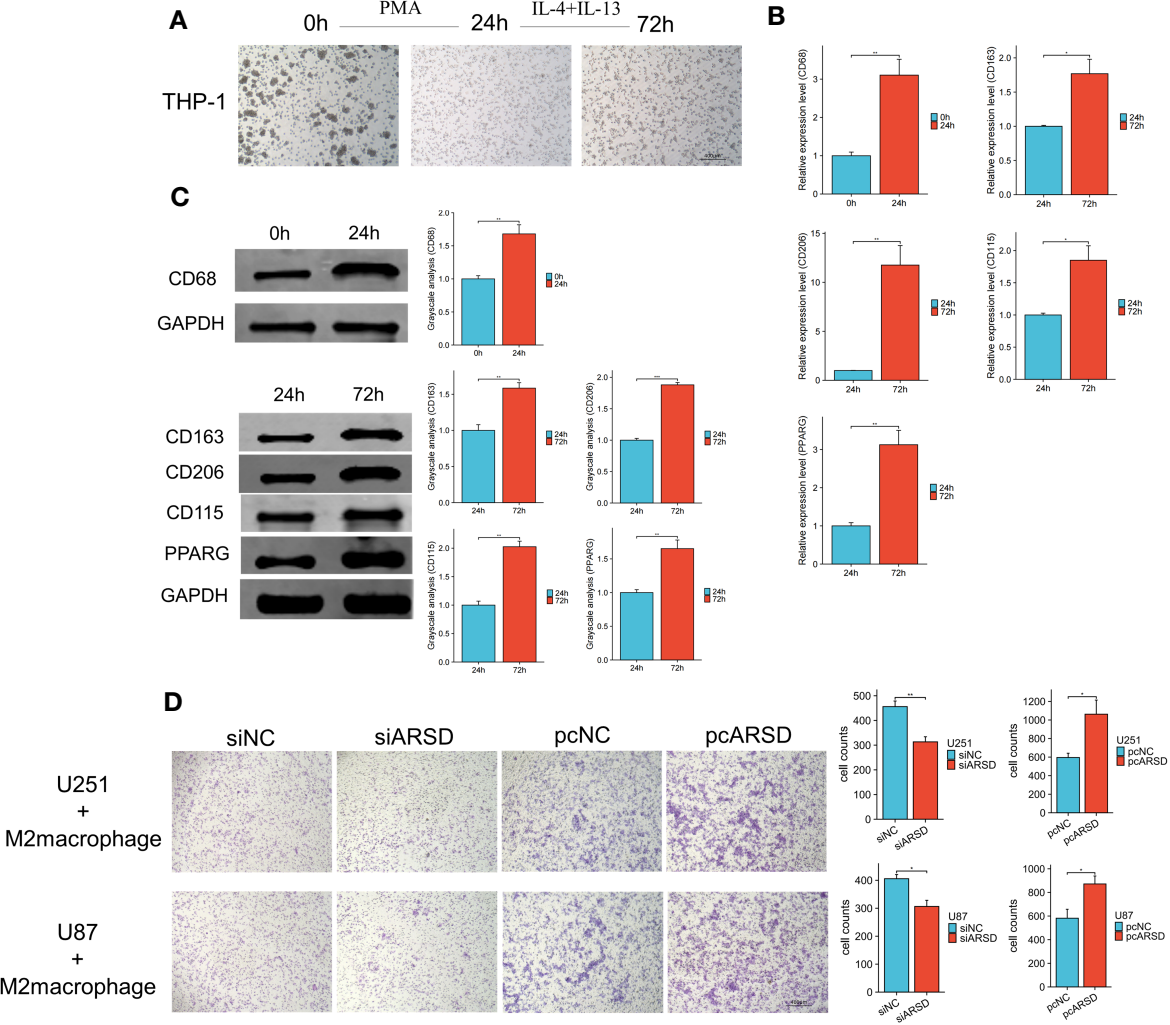
ARSD could enhance the infiltration of M2 macrophages. **(A)** Morphological changes from THP-1 to M2 macrophages. **(B, C)** RT-qPCR and Western blot showed the markers of M2 macrophages (CD68, CD163, CD206, CD115, and PPARG) elevated after induction. **(D)** M2 macrophage infiltration was significantly reduced after ARSD knockdown and increased after ARSD overexpression in glioma cells. * *p* < 0.05 , ** *p* < 0.01, *** *p* < 0.001.

To investigate the relation between ARSD and M2 macrophages infiltration, M2 macrophage infiltration assay was conducted. After using classical inducing methods, we observed the transformation from THP-1 cells to M2 macrophages (**Figure 9A**). RT-qPCR and western blot showed that the markers of M2 macrophages were elevated, such as CD68 CD163, CD206 CD115, PPARG (**Figures 9B, C**). Our results demonstrated that the infiltration of M2 macrophages was obviously weakened after ARSD knockdown, but significantly enhanced after ARSD overexpression (**Figure 9D**).

### ARSD promotes glioma progression through JAK2/STAT3 pathway

**Figure 10:**
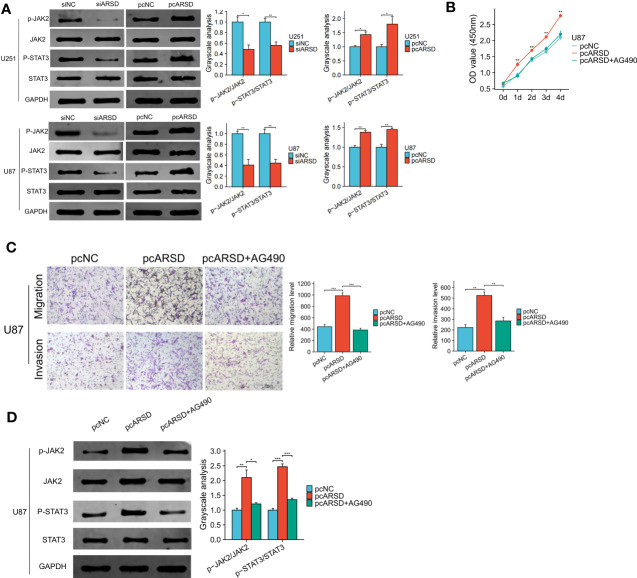
ARSD promote glioma progression through JAK2/STAT3 pathway. **(A)** JAK2/STAT3 pathway was suppressed after ARSD knockdown and activated after ARSD overexpression in glioma cell lines. **(B, C)** AG490 reversed the increased proliferation, migration, and invasion in glioma cell lines caused by ARSD overexpression. **(D)** AG490 reversed the overexpression of p-JAK2 and p-STAT3 in the JAK2/STAT3 pathway caused by ARSD overexpression. * *p* < 0.05 , ** *p* < 0.01, *** *p* < 0.001.

Our bioinformatic analysis revealed a significant association between ARSD and the JAK-STAT signaling pathway. To validate the relation between ARSD and JAK-STAT signaling pathway, we performed several cell experiments. Western blot analysis showed that in U251 and U87 cell lines, p-JAK2/JAK2, and p-STAT3/STAT3 ratios decreased with the downregulation of ARSD, but increased with the overexpression of ARSD (**Figure 10A**). These results suggested that ARSD can promote glioma progression through JAK2/STAT3 pathway. Besides, JAK2/STAT3 pathway inhibitor AG490 was also used to block the activation of this pathway. Using CCK-8 and Transwel, we found the increased proliferation, migration, and invasion of glioma cells induced by ARSD overexpression were reversed upon inhibition of the JAK2/STAT3 signaling pathway with AG490 (**Figures 10B, C**). AG490 was shown to reverse the overexpression of p-JAK2 and p-STAT3 in JAK2/STAT3 pathway resulting from ARSD overexpression, as demonstrated by western blot analysis (**Figure 10D**).

## Discussion

Studies have revealed that the arylsulfatase family might play a role in tumorigenesis and cancer progression, suggested that therapy targeted arylsulfatase family may become a promising strategy for cancer treatment (9). However, the exact mechanisms of arylsulfatases for tumor development were rarely investigated in the past. Our research performed bioinformatic analysis of arylsulfatase family genes in glioma and found that most of these genes expressed the abnormally. Survival analysis showed that ARSD was the prognostic indicator both in TCGA LGG and TCGA GBM. Although it was reported that ARSD was dysregulated in different types of cancer, few research had investigated its prognostic predictive value and potential mechanisms. In this study, we discovered that ARSD was upregulated in glioma and could serve as a novel prognostic predictor. It was also suggested that ARSD could promote glioma progression by regulating cell proliferation and macrophage infiltration, as supported by bioinformatic analysis and cell experiments.

We first investigated the prognostic value of ARSD in glioma in multiple glioma cohorts and found that high expression of ARSD tended to have a short survival time. Given the significant changes in the 5th edition of the WHO Classification of CNS tumors, we performed survival analysis according to the new classification and reached a similar conclusion. ARSD was identified as an independent prognostic factor based on univariate and multivariate Cox analyses. These results exhibited the strong predictive abilities of ARSD for glioma prognosis.

Immune microenvironment analysis suggested that ARSD might regulate the immune phenotype and Cancer immunity cycle, which lead to a suppressive immune microenvironment. Previous studies have indicated that immune cells and stromal cells played a key role in the anti-tumor immune response (25). The immunosuppressive microenvironment in glioma involves a variety of infiltrating immune cells, such as M2 macrophages, Treg cells, neutrophils, and Th2 cells. The findings of our study suggested a positive correlation in ARSD and the immunosuppressive cells levels. Moreover, our results revealed that ARSD was involved in various steps of the Cancer-Immunity Cycle. These findings illustrated that ARSD played a critical role in glioma immune microenvironment.

Macrophages have a crucial role in regulating tumor growth, invasion, and recurrence within the glioma tumor microenvironment (TME). M2 macrophages promote tumorigenesis and progression probably by activating th2-type immune response (26). M2 macrophages may promote tumor progression and poor prognosis by inhibiting CD8+ T cell function (27).Numerous studies have shown that various chemokines, including CCL2, CXCL12, LOX, MCP-3, and M-CSF, are secreted by glioma cells to attract M2 macrophages and change their phenotypes (28). To investigate the relation between ARSD and M2 macrophages infiltration, we performed M2 macrophage infiltration assay and found that the infiltration of M2 macrophages was significantly decreased after knocking down ARSD. It suggested that ARSD might affect the process of macrophage infiltration in glioma. Based on these results, targeting ARSD may represent a promising approach for preventing the infiltration of macrophages by glioma cells. However, an increasing number of studies revealed the critical roles of tumor microenvironment in immunosuppression. Considering the lack of tumor microenvironment *in vitro*, our conclusions also need to be verified using patient-derived tumor cells and *in vivo* experiment.

Prior studies have demonstrated a significant involvement of the JAK2/STAT3 pathway in the progression and development of human malignant tumors (29). Upon nuclear translocation, phosphorylated STAT3 regulates the transcription of specific target genes that govern fundamental physiological processes. Consequently, this process results in aberrant gene expression involved in cell differentiation, proliferation, and apoptosis, such as Bcl-XL and c-Myc, which promote cell proliferation and malignant transformation (30). Enrichment analysis showed that ARSD was significantly related to JAK2/STAT3 pathway. Cell experiment showed that modulation of the JAK2/STAT3 pathway by CTR9 was found to promote proliferation, migration, and invasion of glioma cells. Besides, our results further revealed that the upregulation of ARSD, which promoted glioma cell proliferation, migration and invasion, could be effectively inhibited by the JAK2/STAT3 pathway inhibitor AG490.

In this research, we fully explored the prognostic value of ARSD and investigated the potential mechanisms. Based on the new classification of glioma in WHO, we found that ARSD was a risk factor for overall survival in various subtypes of glioma, and has good predictive value for glioma prognosis. With the combination of bioinformatic analysis and experimental validation, we identified that ARSD can promoted glioma progression by regulating JAK2/STAT3 pathway and M2 macrophage infiltration. However, there are still some shortcomings in our research. First, the glioma cell lines used in this research were purchased from commercial vendors. Recent studies have shown that patient-derived tumor cells had some advantages compared to the cell lines from commercial vendors, such as better representation of tumor heterogeneity, retention of original genomic and epigenomic features. Our conclusions also need to be verified using patient-derived glioma cells and *in vivo* experiments. Second, it is necessary to conduct further studies with prospective and multicenter clinical cohort, which make our prognostic analysis more convincing.

## Conclusion

This study showed that ARSD could serve as a predictive factor for glioma prognosis. High level of ARSD promoted the development of glioma by regulating M2 macrophage infiltration and JAK2/STAT3 pathway. Our findings provide new understandings about the molecular mechanisms and target therapy in glioma.

## Data availability statement

Publicly available datasets were analyzed in this study. This data can be found here: 1.UCSCXenaShiny (https://hiplot-academic.com/advance), WOS: 000743380100030. 2. Gliovis (http://gliovis.bioinfo.cnio.es/), WOS: 000393892800023. 3. (https://cibersortx.stanford.edu), MEDLINE:29344893.

## Ethics statement

This study was approved by the Research Ethics Committee of the Second Hospital of Hebei Medical University. Approval letter No. 2023-R044. Written informed consent for participation was not required for this study in accordance with the national legislation and the institutional requirements.

## Author contributions

ZS mainly responsible for manuscript writing and data analysis. ZS, ZiZ, SiZ, and ShZ conducted experiments. QJ, ZaW, BS, and ZiW collected the samples and clinical data. ZS created tables, graphs, and figures. ZMZ conceived the idea for the project, revised the paper, and received funding for the project. All authors contributed to the article and approved the submitted version.
