# Supplementary material for: Arylsulfatase D is a prognostic biomarker that promotes glioma cells progression through JAK2/STAT3 pathway and M2 macrophage infiltration
Source: Front Oncol. 2023 Sep 12;13:1228426. doi: 10.3389/fonc.2023.1228426 (PMC10521731; doi:10.3389/fonc.2023.1228426)
Supplement: Supplementary file 1 [file DataSheet_1.docx]

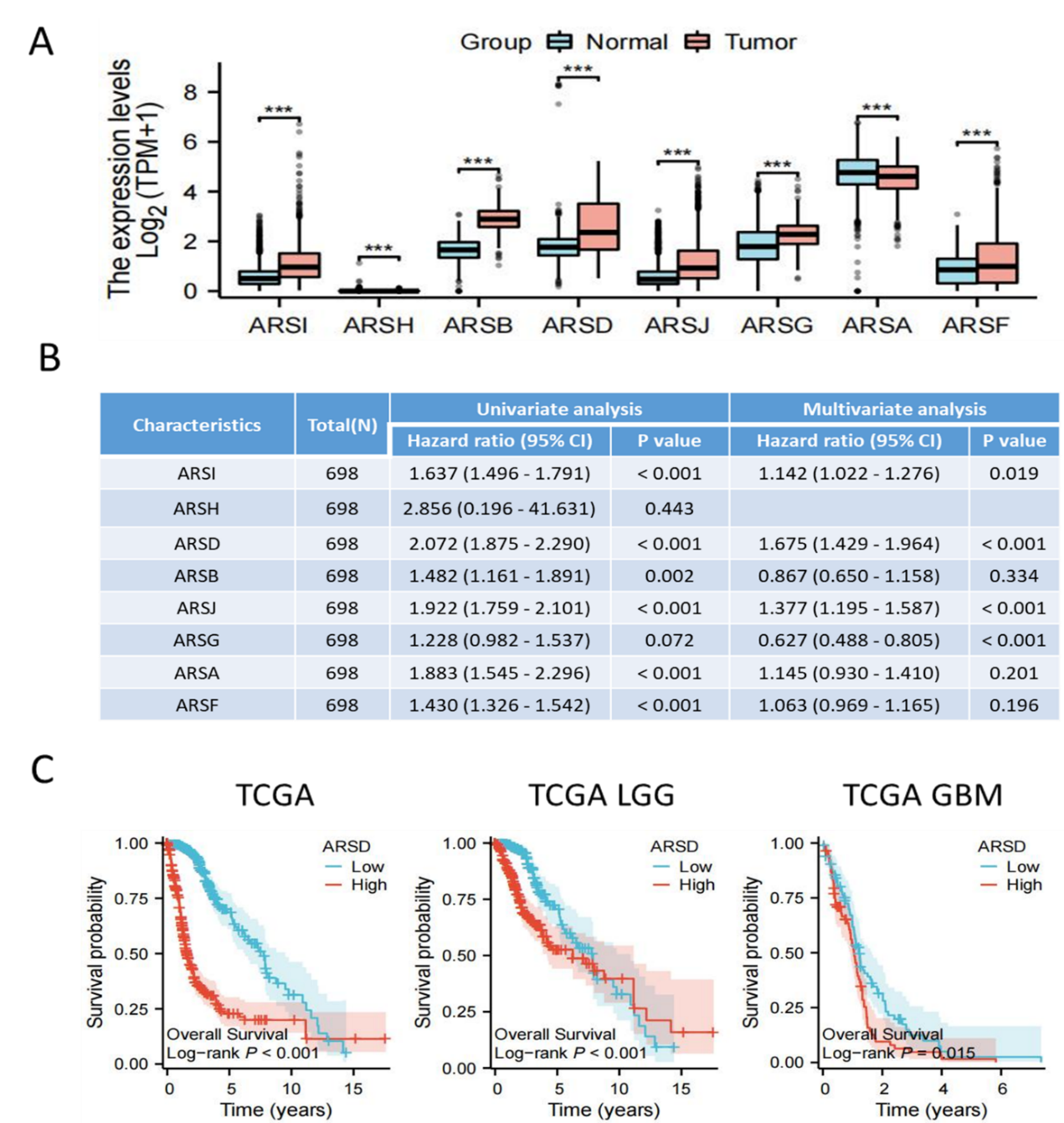


**Supplemental Figure 1. Analysis of the arylsulfatase family in TCGA glioma cohort.** (A) Expression of arylsulfatase family in TCGA. (B) Univariate and multivariate analysis of arylsulfatase family in TCGA glioma cohort. (C) Survival analysis showed that high expression of ARSD tented to have a short OS in both TCGA LGG and TCGA GBM.


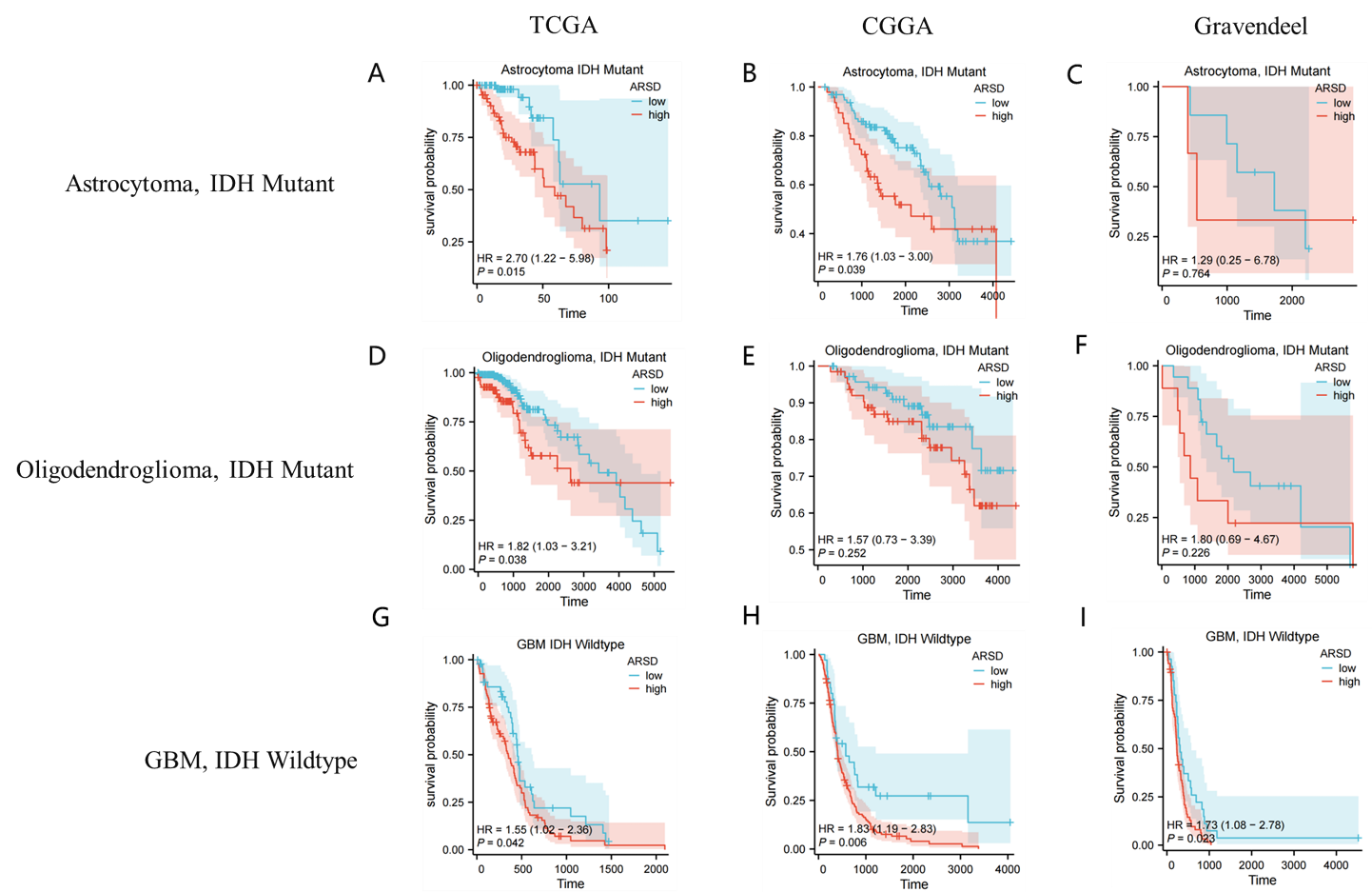


**Supplemental Figure 2. Survival analysis of ARSD in different glioma subtypes in TCGA, CGGA and Gravendeel cohort.** (A-C) Astrocytoma, IDH Mutant; (D-F) Oligodendroglioma, IDH Mutant; (G-I) GBM, IDH Wildtype.


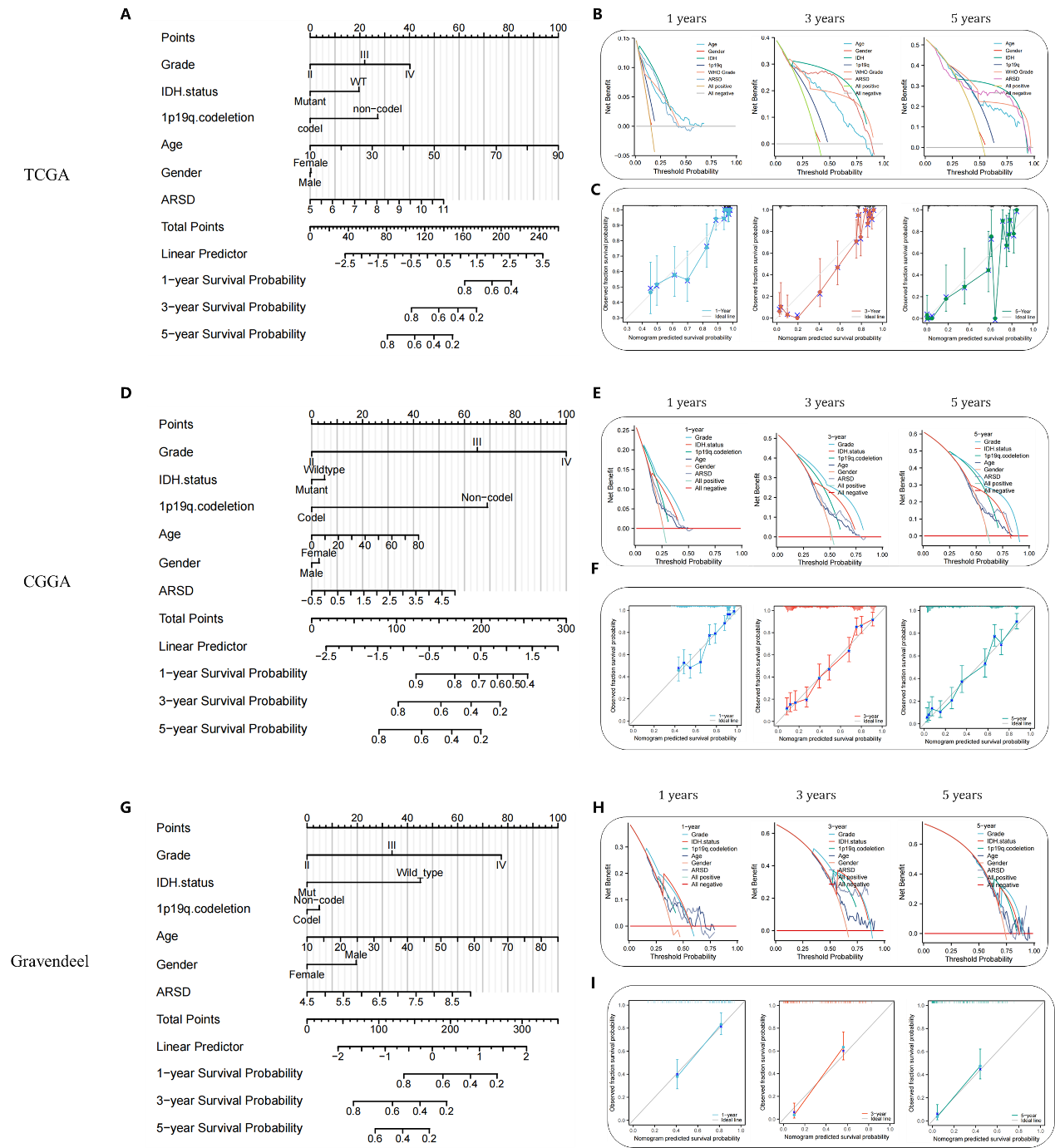


**Supplemental Figure 3. Nomogram establishment.** (A, D, G) Nomogram for prognostic prediction of glioma in TCGA, CGGA and Gravendeel. (B, E, H) The performance of the TCGA, CGGA, and Gravendeel nomograms in clinical decision-making for 1-year, 3-year, and 5-year survival was evaluated using decision curves. (C, F, I) Calibration curves revealed the predictive value of this nomogram at 1, 3, and 5 years.
